# Track Structure-Based Simulations on DNA Damage Induced by Diverse Isotopes

**DOI:** 10.3390/ijms232213693

**Published:** 2022-11-08

**Authors:** Pavel Kundrát, Werner Friedland, Giorgio Baiocco

**Affiliations:** 1 Department of Radiation Dosimetry, Nuclear Physics Institute, Czech Academy of Sciences, Na Truhlářce 39/64, 180 00 Prague, Czech Republic; 2 Institute of Radiation Medicine, Helmholtz Zentrum München, GmbH—German Research Center for Environmental Health, Ingolstädter Landstr. 1, 85764 Neuherberg, Germany; 3 Radiation Biophysics and Radiobiology Group, Physics Department, University of Pavia, 27100 Pavia, Italy

**Keywords:** ionizing radiation, isotopes, track structure, DNA damage, Monte Carlo simulations, analytical functions

## Abstract

Diverse isotopes such as ^2^H, ^3^He, ^10^Be, ^11^C and ^14^C occur in nuclear reactions in ion beam radiotherapy, in cosmic ray shielding, or are intentionally accelerated in dating techniques. However, only a few studies have specifically addressed the biological effects of diverse isotopes and were limited to energies of several MeV/u. A database of simulations with the PARTRAC biophysical tool is presented for H, He, Li, Be, B and C isotopes at energies from 0.5 GeV/u down to stopping. The doses deposited to a cell nucleus and also the yields per unit dose of single- and double-strand breaks and their clusters induced in cellular DNA are predicted to vary among diverse isotopes of the same element at energies < 1 MeV/u, especially for isotopes of H and He. The results may affect the risk estimates for astronauts in deep space missions or the models of biological effectiveness of ion beams and indicate that radiation protection in ^14^C or ^10^Be dating techniques may be based on knowledge gathered with ^12^C or ^9^Be.

## 1. Introduction

The biological effects of ionizing radiation vary hugely with radiation quality, from sparsely ionizing photons and electrons to protons and densely ionizing heavier ions such as carbon or iron ions. This has been addressed in dedicated radiobiological studies investigating endpoints such as DNA damage induction, its repair, induction of chromosomal aberrations, mutations or cell killing; reviewed, for example, by [1,2,3,4,5]. Modelling and simulation tools, especially Monte Carlo track structure simulations, have succeeded in explaining the observed trends and linking them to the initial physical processes that lead to distinct track structures, as well as subsequent chemical processes of radical formation, reactions, and attack on cellular DNA [6,7]. In the vast majority of both experimental and modelling studies with ions, the naturally most abundant isotope of the given element was used, typically in its fully ionized form, e.g., protons (^1^H^+^ ions) or ^12^C^6+^ ions. This case also corresponds to most applications of ion beams, e.g., proton or carbon beams in radiation therapy for the treatment of cancer [8,9].

Nevertheless, other less abundant and even unstable isotopes are of interest, in fields as diverse as radiotherapy, space research or dating various objects or materials using radiocarbon (^14^C), ^10^Be or other isotopes. In radiotherapy with ion beams, diverse isotopes frequently occur as products of nuclear reactions in projectile or target fragmentation. Although electronic interactions are the predominant mechanism of energy loss, leading to the sharp dose-deposition pattern of the Bragg peak, products of nuclear reactions modulate the shape of depth-dose curves and form a fragmentation tail, which has to be considered in treatment planning. For instance, for a therapeutic 200 MeV/u ^12^C beam, about 30% of primary particles undergo nuclear reactions along its ~8.6 cm penetration depth in tissue [10]. In these reactions, mainly protons, deuterons (^2^H nuclei), alpha particles (^4^He nuclei) and also ^10^C and ^11^C nuclei are produced [11,12], with a wide range of energies, essentially up to that of the primary particles. In-beam ^11^C production can be utilized in beam delivery monitoring with positron emission tomography [12]. Further, therapeutic proton beams produce secondary ^2^H, ^3^H, ^3^He, ^4^He and other fragments, which may need to be accounted for in treatment planning [13]. The direct usage of ^3^He, ^10^C or ^11^C beams in radiotherapy has also been proposed [14,15]. Regarding space research, a wide variety of elements and their isotopes with energies occasionally exceeding 1 GeV/u are present in cosmic rays or are produced when cosmic rays get modulated by spacecraft shielding materials or planetary atmospheres [16,17,18]. Potential long-term effects of space radiation exposure comprise the induction of cancer, circulatory diseases or damage to the central nervous system, including cognitive defects. In fact, radiation protection considerations are among the key factors hindering long-term Moon or Mars missions [19]. Finally, in accelerator mass spectrometry (AMS) techniques used for dating various materials of organic origin, architectural remains or geological samples with wide applications, diverse isotopes such as ^13^C, ^14^C, ^10^Be or ^26^Al are intentionally accelerated to energies in MeV and sub-MeV ranges [20]. Also in this field, radiation safety measures require knowledge of the biological effects of accelerated isotopes.

Despite the interest in the isotope-specific biological effects of radiation for all of the abovementioned scenarios, only a few radiobiological studies have directly addressed this issue. These include experiments on DNA damage and cell killing induced by deuterons compared with protons, or ^3^He compared with ^4^He ions [21,22,23,24]. These studies indicated that, at the investigated energies of a few MeV/u, biological effects of deuterons do not differ from those of protons at the same linear energy transfer (LET), and the same holds for ^3^He compared with ^4^He. In fact, deuterons have often been used in radiobiological research to extend the ranges of LET achievable with protons, and ^3^He to avoid technical issues with ^4^He [25,26,27]. Also, existing biophysical models of radiation effects have assumed that radiobiological effects are isotopically independent [28], or have not dealt with diverse isotopes at all [29,30,31,32,33]. To our knowledge, the biological effects of diverse isotopes have not been systematically modelled by track structure-based simulations either. Recently, we have presented comprehensive results on DNA damage patterns induced by ions from H to Ne at energies from 0.5 GeV/u down to their stopping, obtained with PARTRAC biophysical simulations [34]. We have provided analytical formulas that allow using these simulation results as a method to extend macroscopic transport codes to biological endpoints [35,36]. However, these studies were performed for the naturally most abundant isotope of each element only. In this work we thus extend our previous simulations to [34] explicitly consider the diverse isotopes of H, He, Li, Be, B and C, started with energies of 0.25–512 MeV/u (for H and He, 0.1–512 MeV/u) from a circular source tangential to the cell nucleus. The induction of DNA damage is reported in terms of DNA single- and double-strand breaks (SSB, DSB), DSB sites and clusters [6,34].

## 2. Results

**Figure 1:**
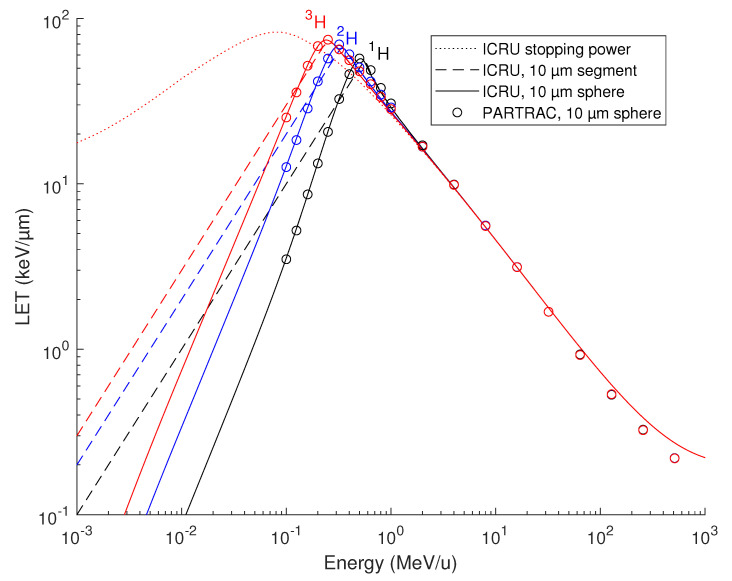
Energy deposited to the cell nucleus volume by protons ^1^H (black), deuterons ^2^H (blue) and tritons ^3^H (red) simulated with PARTRAC, compared with ICRU stopping power values. PARTRAC simulations for a 10 µm diameter sphere are plotted in dependence on the particles’ starting energy (symbols). ICRU stopping powers are shown either as local values at the given particle energy (dotted line) or integrated over a 10 µm track segment (dashed lines) or a 10 µm diameter sphere (solid lines).

Energy depositions to the spherical lymphocyte nucleus by protons ^1^H, deuterons ^2^H and tritons ^3^H simulated with PARTRAC are presented in Figure 1 (symbols), where the nucleus-averaged LET is shown in dependence of the particles’ initial energy. For simplicity, the terms protons, deuterons and tritons denote the energy-dependent mixtures of charged states, e.g., ^2^H^+^ and ^2^H^0^. For comparison, the ICRU stopping power for protons in liquid water [37,38] is also presented in Figure 1. Here, the ICRU stopping power tables were used (i) to plot the local stopping power of hydrogen ions at the given energy per nucleon, which is identical for all 3 isotopes (dotted line); (ii) to calculate the LET integrated over a 10 µm track segment (relevant, e.g., for particles impinging on a cylindrical cell nucleus, parallel with its axis; dashed lines); or (iii) over a 10 µm diameter sphere (solid lines), with the given starting energy of the particles. As both the integrated stopping power values and PARTRAC simulations show, at low energies (< 1 MeV/u), the energy deposited to the nucleus (either spherical or cylindrical), and hence the nucleus-averaged LET, vary among the isotopes due to the kinematic effects: Deuterons, for instance, possess the same stopping power as protons of the same velocity, i.e., of the same energy per nucleon. Deuterons thus lose their energy at the same absolute rate as protons. However, as deuterons possess approximately twice the mass of protons, their energy (absolute, not per nucleon) is about twice as high, and the identical absolute energy loss actually means half the relative rate and translates into a longer range. Consequently, the energy deposited to the nucleus largely varies with the neutron number. For instance, 0.1 MeV/u deuterons deposit, to the spherical cell nucleus, about three times more energy than protons, and tritons twice more than deuterons. Above 1 MeV/u, there are virtually no differences between the energy deposited to the nucleus by protons, deuterons and tritons, since the energy loss over the track segment is sufficiently small compared with the particles’ energy, and hence the kinematic effects are negligible. Overall, the PARTRAC simulations on nucleus-averaged LET calculated from the energy deposited in the spherical nucleus (symbols) nicely match the integrated ICRU stopping powers for the same geometry (solid lines). The agreement is very good up to about 30 MeV/u. Above this value, the LET given by the energy deposited to the nucleus is somewhat smaller than the stopping power. This is due to the generated high-energy secondary electrons carrying a part of the energy away from the nucleus, both laterally and longitudinally, so that the stopping power fails to correctly represent the energy actually deposited to the nucleus.

**Figure 2:**
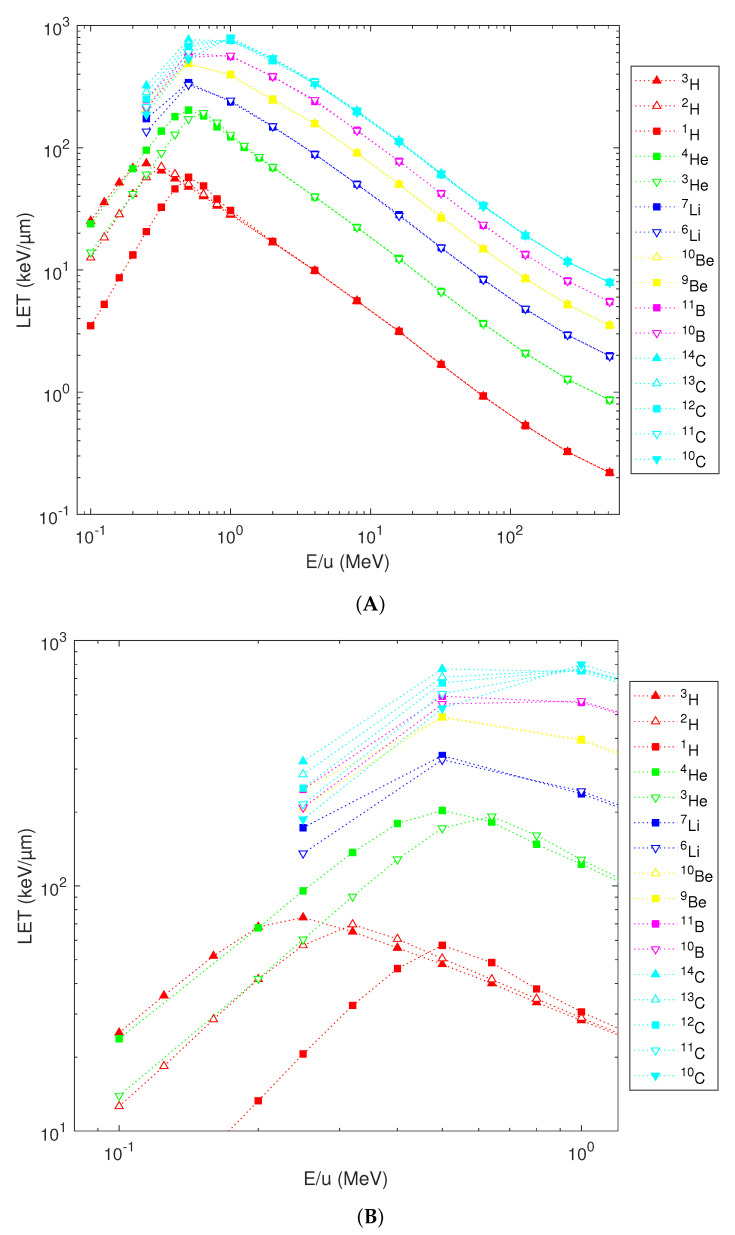
Simulated energy depositions to a 10 µm diameter sphere by H, He, Li, Be, B and C isotopes in dependence on their starting energy (panel (**A**): whole simulated energy range, panel (**B**): detailed view of the low-energy region). The results for the naturally most abundant isotopes are depicted by squares, those for isotopes having less or more neutrons by downward or upward-heading triangles; empty or full triangles if differing from the most abundant isotope by one or two neutrons, respectively. Lines are a guide to the eye.

As shown in Figure 2, the nucleus-averaged energy deposition is virtually independent of the neutron number at energies above 1 MeV/u also for species with a higher atomic number. Variations among isotopes of a given element occur below this value, but get reduced with an increasing atomic number. For instance, at 0.25 MeV/u, while ^2^H deposits 2.78-times higher energy to the cell nucleus than protons do, between ^12^C and ^11^C this ratio is just 1.16.

**Figure 3:**
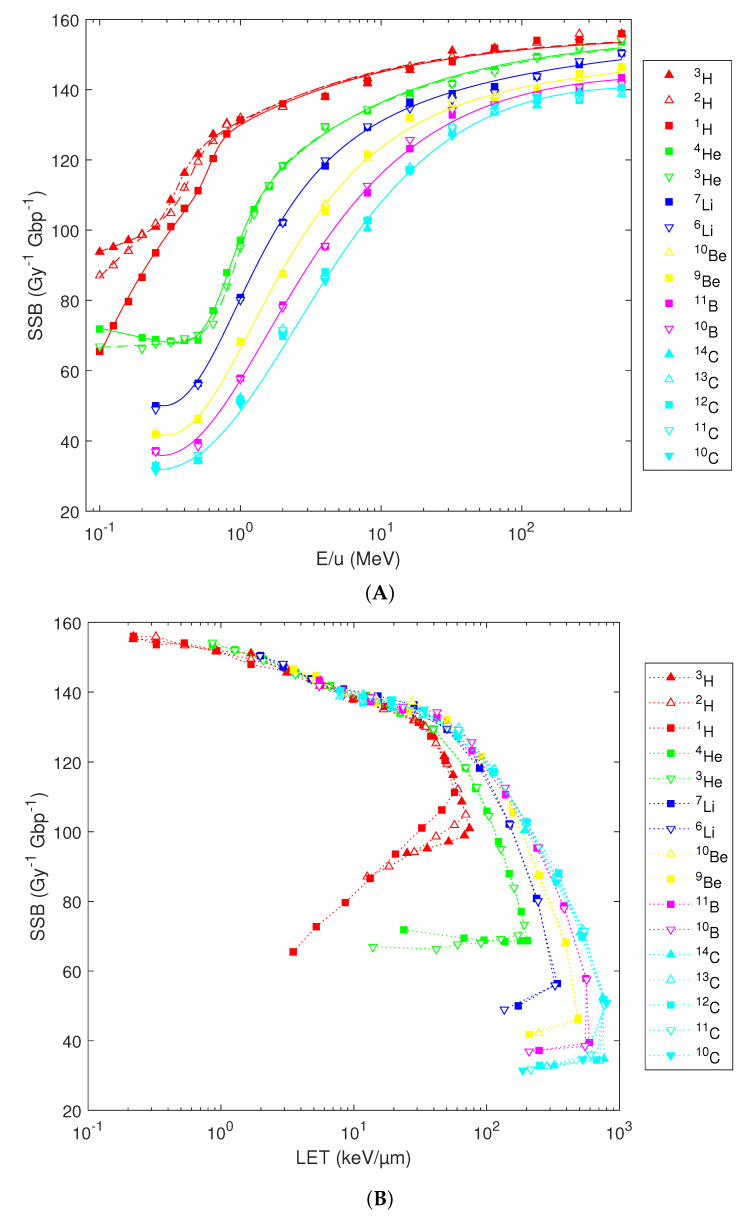
Simulated yields of DNA single-strand breaks (SSB) by H, He, Li, Be, B, or C isotopes, plotted in dependence on the particles’ starting energy (panel (**A**)) or nucleus-averaged LET (panel (**B**)). Symbols depict the PARTRAC simulation results, lines in panel A reflect their analytical fits using Equation 1 with parameters listed in Table 1, while lines in panel B, are to guide the eye only.

Since the energy deposition to the cell nucleus does depend on the number of neutrons, the same can be expected for the induction of DNA damage. When normalized to unit dose, i.e., presented in terms of yields per Gy per Gbp, DNA damage is isotope-specific only for the lowest atomic numbers (especially hydrogen and helium isotopes) and at energies below 1 MeV/u. As shown in Figure 3A, the yields of DNA single-strand breaks (SSB) generally decrease with an increasing atomic number and with decreasing energy, from about 140–160 per Gy per Gbp above 100 MeV/u down to 30–90 SSB per Gy per Gbp at the particles’ initial energy of 0.1–0.25 MeV/u, in agreement with previous results [34]. Remarkable differences are predicted for low-energy hydrogen isotopes with for instance, 0.1 MeV/u deuterons (or tritons) induce 30% (or 40%) more SSB per Gy per Gbp than protons started with the same velocity. Note that the yields per track increase much more than the yields per Gy, as the latter are corrected for the increased LET. These variations rapidly diminish with increasing initial energy or atomic number. Consequently, while separate analytical fits are needed for H and He isotopes, a single fit per element is sufficient for isotopes of heavier elements. The fits based on Equation (1) are depicted in Figure 3A by lines; the corresponding fit parameters are listed in Table 1.

The separation of SSB yields with atomic number predominantly follows from differences in ionization density as captured by the nucleus-averaged LET [34]. Indeed, when plotted in dependence on the LET, this variability gets reduced; the simulation results for all isotopes follow a unique trend for LET < 10 keV/µm (Figure 3B). The reduction in SSB yields with decreasing energy (increasing LET) is largely due to the enhanced clustering of energy deposition events and hence the induction of more complex types of DNA damage, including DNA double-strand breaks (DSB). A drawback of the LET representation is that particles at two distinct energies, on proximal and distal parts of the Bragg peak, may possess an identical LET but a different efficiency in SSB induction, leading to hooks in the plots. Consequently, the simulation results cannot be fitted by a single function over the whole simulated range. Obviously, variations among isotopes at low energies also remain present in the LET-dependent plots.

**Figure 4:**
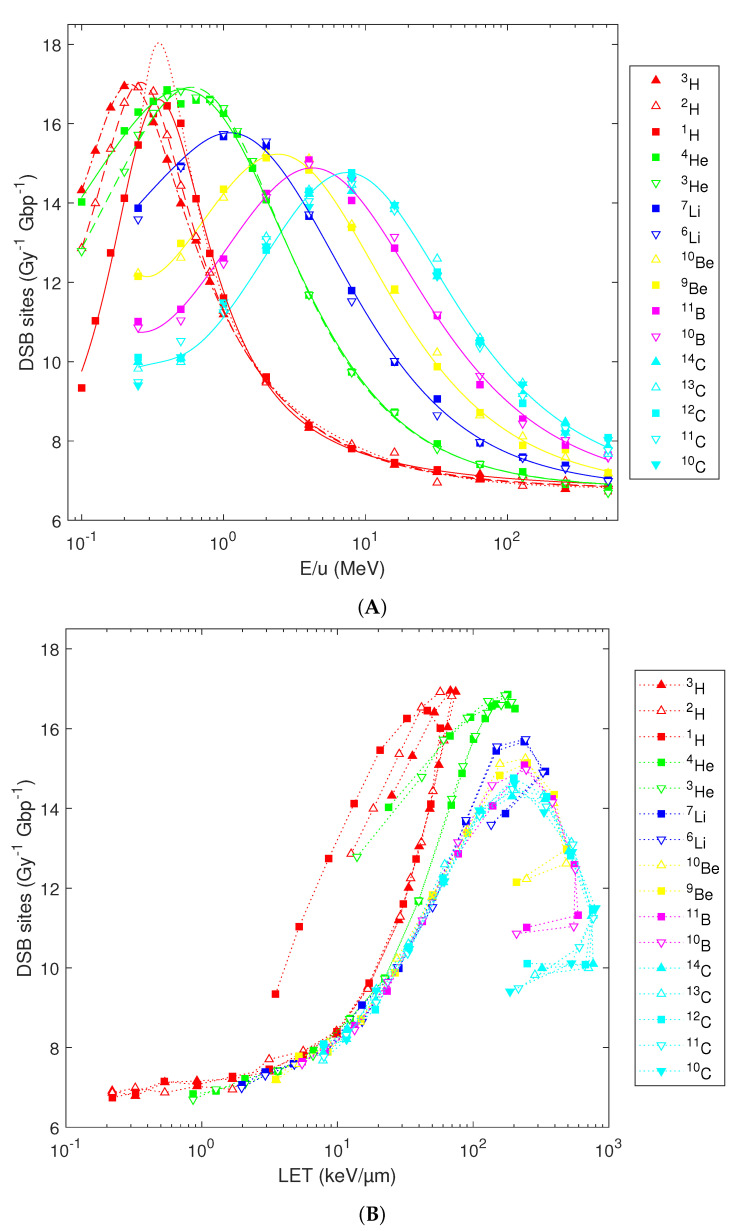
Simulated induction of DSB sites in human lymphocytes by isotopes from H to C, plotted in dependence on (**A**) the particles’ starting energy or (**B**) the nucleus-averaged LET. Symbols: PARTRAC simulations, lines: analytical fits ((**A**); see text for details) or to guide the eye only (**B**).

The predicted DSB induction shows multiple trends. First, the yields of DSB sites (i.e., genomic sites containing either an isolated or clustered DSB) possess a bell-shaped dependence on the particles’ initial energy (Figure 4A). With an increasing atomic number, the maximal yields get slightly reduced and shift towards higher energies, from 16.5–16.9 DSB sites per Gy per Gbp for hydrogen isotopes at 0.2–0.4 MeV/u to 14.3–14.8 DSB sites per Gy per Gbp for carbon isotopes at 8 MeV/u. Notable differences are predicted for low-energy hydrogen isotopes, e.g., with 0.1 MeV/u deuterons (or tritons) inducing 40% (or 56%) more DSB sites per Gy per Gbp than protons starting with the same energy per nucleon. However, the variations among isotopes diminish with increasing energy as well as with increasing atomic number and get marginal for energies above 1 MeV/u or ions heavier than helium. Consequently, the previously reported analytical formulas for DSB sites [36] can be used for elements heavier than He (blue, yellow, magenta and cyan lines). However, for H or He isotopes, the use of refined formulas based on Equation 1 is recommended (solid, dashed and dash-dotted red and green lines in Figure 4A, parameters listed in Table 1). These refined formulas reproduce the simulation results over the whole studied energy range and reflect the variations among H or He isotopes at energies below 1 MeV/u; above 1 MeV/u, the refined and original formulas provide almost identical results. For low-energy protons, the refined formulas also remove the issue that the original one (dotted red line) overestimated the peak damage induction at 0.3–0.4 MeV/u, as revealed by the dense grid of starting energies used in the present study.

As was the case for SSB, most of the variability with the atomic number in the yields of DSB sites diminishes when the simulation results are plotted in dependence on the LET (Figure 4B). At the same LET, H isotopes are more effective in inducing DSB sites than He, and He is more effective than heavier ions. Variations among isotopes remain present, and LET-dependent plots show hooks as discussed for SSB.

**Figure 5:**
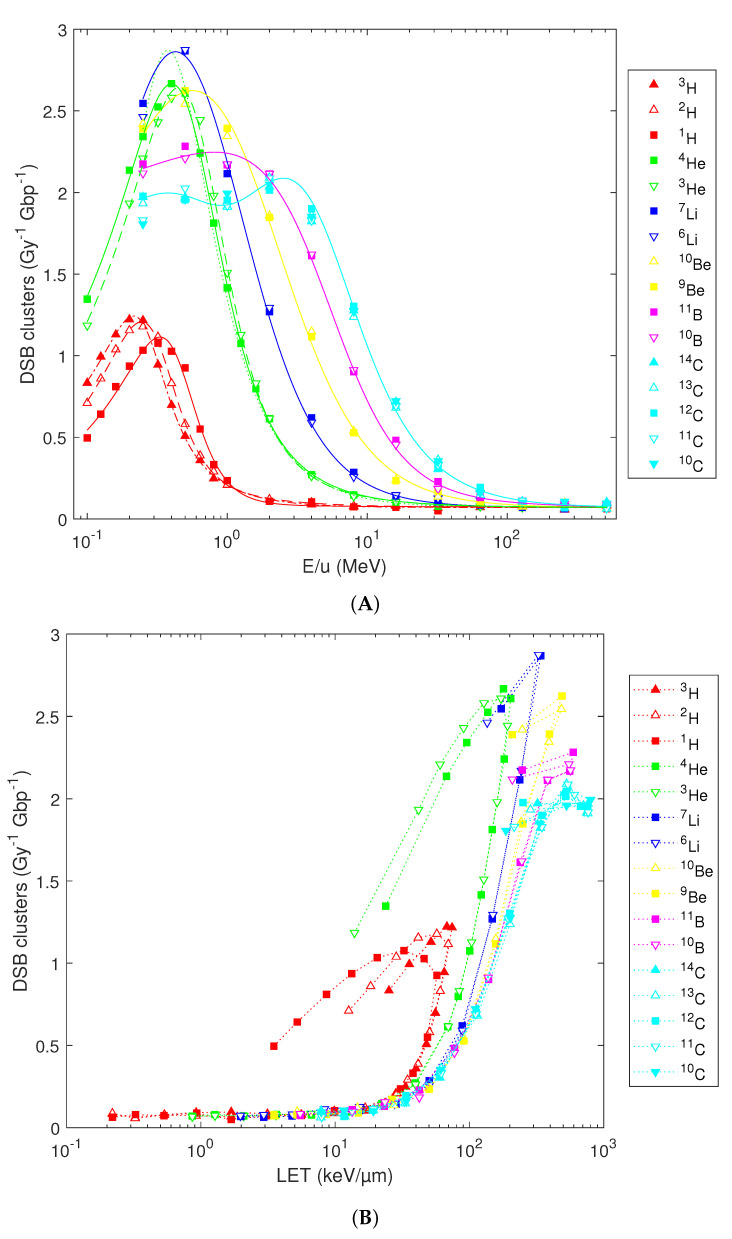
Predicted yields for DSB clusters (symbols) by diverse isotopes in dependence on their starting energy (**A**) or nucleus-averaged LET (**B**). Lines show the analytical fits (**A**); see text for details) or serve to guide the eye only (**B**).

The results of simulations on the induction of DSB clusters are presented in Figure 5. Compared with DSB sites, DSB clusters are much less frequent: Their predicted yields range from 0.05 per Gy per Gbp for high-energy species to 1.1–1.2 per Gy per Gbp for low-energy H and 2.0–2.9 per Gy per Gbp for heavier ions (Figure 5A). Interestingly, Li isotopes around 0.5 MeV/u are the most efficient species as far as the numbers of induced DSB clusters per Gy are concerned. Again, refined analytical formulas are needed for capturing the simulation results for H and He isotopes below 1 MeV/u (solid, dashed and dash-dotted red and green lines in Figure 5A, formulas based on Equation 1 with parameters listed in Table 1). The refined formulas are valid over the whole of the studied energy range. They remove the overestimation by the previously published formula [36] of DSB cluster yields by low-energy He particles (dotted green line), revealed by the dense energy grid used in the present work. For energies above 1 MeV/u, the original and refined formulas provide almost identical results. Likewise, the previously published formulas can be applied to isotopes of Li, Be, B and C (blue, yellow, magenta and cyan lines). Similarly to the other DNA damage classes, when the simulation results on DSB clusters are plotted versus LET, the variability with atomic number gets reduced, but the isotopic specificity remains present and hooks appear as for the aforementioned DNA damage classes (Figure 5B).

**Figure 6:**
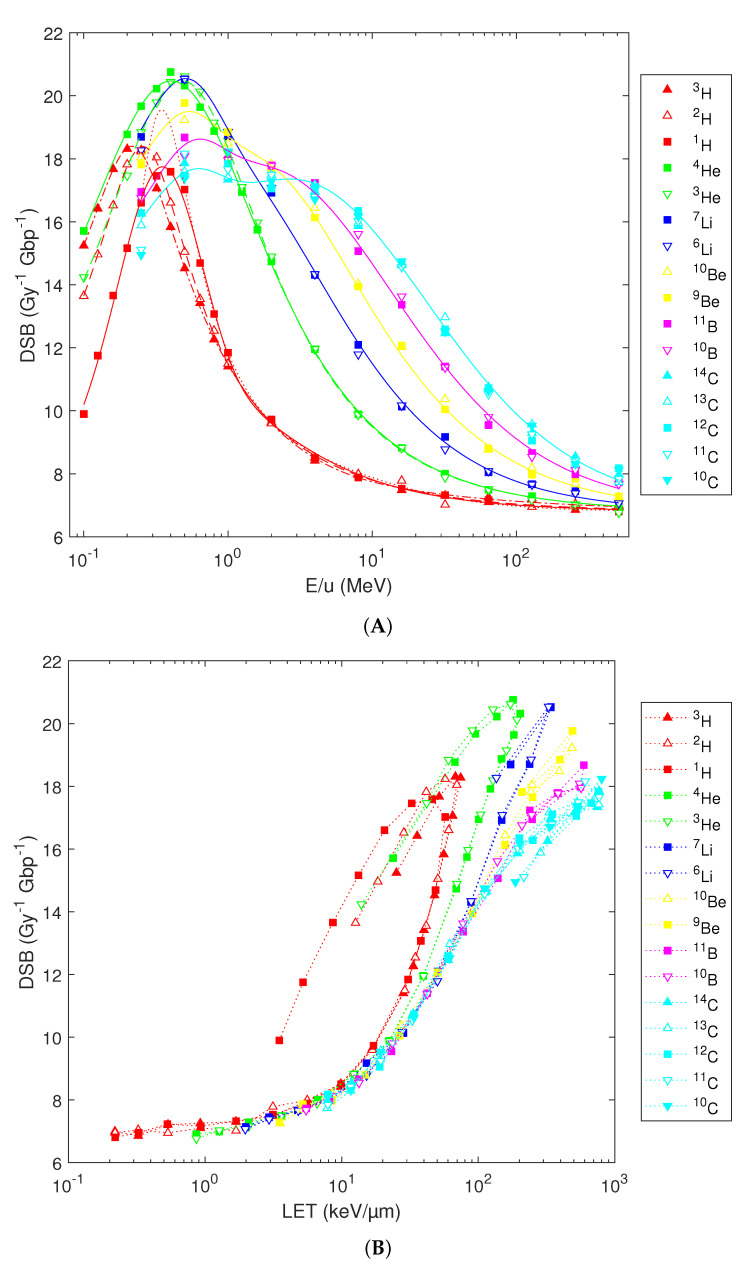
Predicted total DSB numbers induced by isotopes from H to C in human lymphocytes. Simulation results (symbols) plotted in terms of particles’ starting energy (**A**) or nucleus-averaged LET (**B**). Lines show analytical fits (**A**); see text for details) or are presented to guide the eye only (**B**).

When the total DSB yields are considered, counting isolated DSB as single ones and each DSB in a cluster separately, the trends combine those of DSB sites and clusters (Figure 6A). He and Li at 0.4–0.5 MeV/u turn out to be the most effective species. Isotope-specific results are obtained for H and He below 1 MeV/u. Refined fit formulas shall thus be used in this region (green and red lines in Figure 6A, fits based on Equation 1 with parameters listed in Table 1). The previously published fits [36] remain applicable at higher energies or for heavier elements. Plotting the results in terms of the nucleus-averaged LET partially explains the observed variability, but brings in hooks; isotopic specificity for low-energy H or He remains present (Figure 6B).

**Figure 7:**
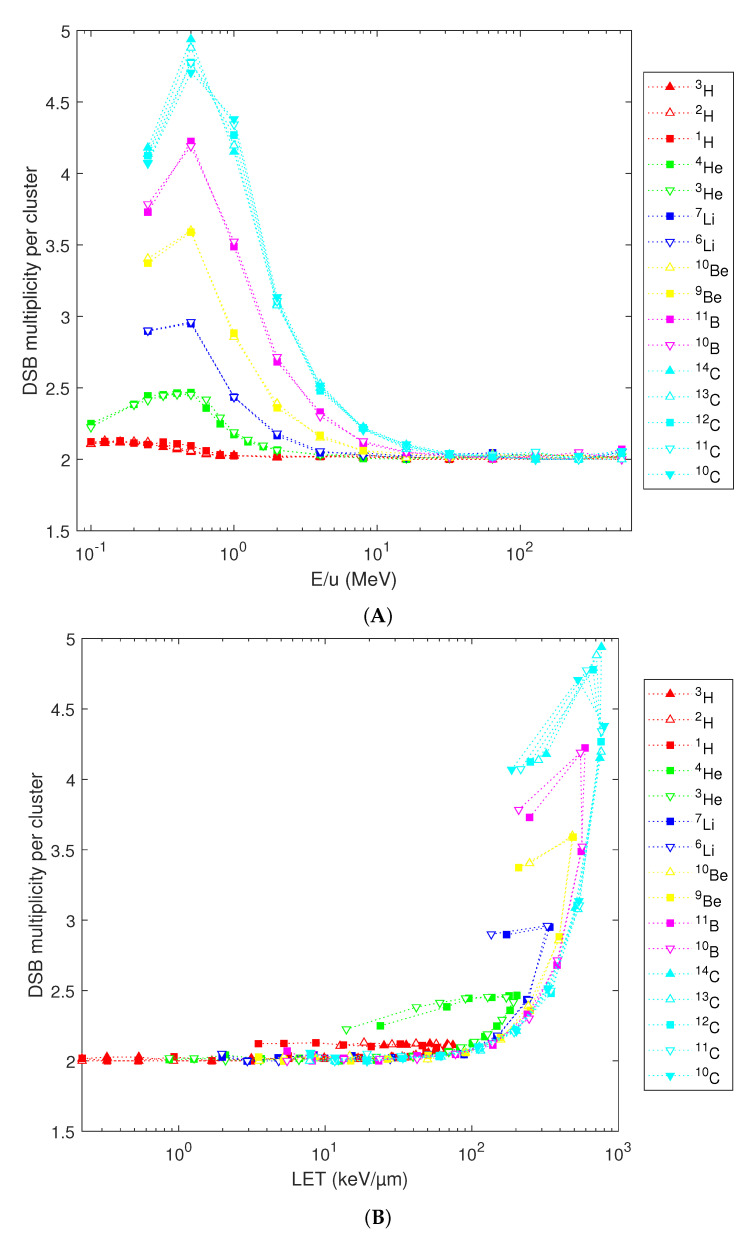
Predicted mean multiplicity of DSB per cluster (symbols), plotted for diverse isotopes in terms of their starting energy (**A**) or nucleus-averaged LET (**B**). Lines are to guide the eyes only.

Finally, simulation results on mean cluster multiplicity (i.e., the mean number of DSB in a cluster) are presented in Figure 7, in dependence on energy per nucleon (panel A) or LET (panel B). Compared with heavier elements, hydrogen isotopes not only induce fewer DSB clusters (Figure 5A), but these typically consist of two DSB only, with a mean cluster multiplicity of 2.1 even for energies below 1 MeV/u, while clusters induced by 0.5 MeV carbon isotopes are on average composed of almost five DSB. Differences among diverse isotopes of the same element are marginal only.

## 3. Discussion 

Dedicated track structure-based simulations of DNA damage induction were performed for isotopes of H, He, Li, Be, B and C, with energies from 0.5 GeV/u down to stopping. The simulations were based on the PARTRAC biophysical tool. Cross sections for electromagnetic interactions were assumed to depend on the atomic number but be independent of the neutron number. While dedicated cross sections were used for hydrogen and helium isotopes, explicitly considering distinct charge states, those for lithium, beryllium, boron and carbon were scaled from the hydrogen of the same velocity, using the effective charge concept. Nuclear reactions within the cell nucleus were neglected, as their relevance over the studied scale of 10 µm is very limited.

The simulation setup consisted of a circular particle source directly touching the spherical nucleus model of a human lymphocyte. Therefore, especially for high-energy ions, the conditions of electronic equilibrium were not fulfilled. Our previous simulations have shown that while this leads to notably reduced doses to the nucleus at high energies (by as much as 20%) compared to electronic equilibrium conditions, the yields of DNA damage per Gy are almost identical in both cases, with differences up to a few percent [39].

DNA single- and double-strand breaks (SSB, DSB), DSB clusters and DSB sites (including either isolated or clustered DSB) were scored. With decreasing initial particle energy, their ionization density quantified by the LET (here defined at the cell nucleus scale) increases and so does the induction of DSB and their clusters, while the induction of SSB decreases. Only at very low energies do DSB and cluster yields decrease with further decreasing energy. These results are in agreement with previous simulations. Comparison with experimental data is not straightforward, as experimental detection limits have to be taken into account. For instance, as presented previously [6], the simulated DSB yields by high-LET ions (about 15–20 DSB per Gy per Gbp) appear much higher than the measured values of 5–10 DSB per Gy per Gbp, or merely 3–6 DSB per Gy per Gbp for protons, deuterons, ^3^He and ^4^He, as reported in [24]. However, when only DSB associated with DNA fragments within the detection limits (e.g., 5 kbp–5.7 Mbp) are considered, the simulation results agree with the experiments [6,34], not just for the total yields, but also for the fragmentation patterns which were previously benchmarked against the data [6]. The additional DSB predicted by the simulations are associated with very short fragments, which are likely to be hardly repairable but remain undetected in classical DNA damage experiments. To limit the computational expenses, a dedicated fragmentation analysis was not performed in the present study.

Different isotopes of a given element at a given velocity (and hence energy per nucleon) possess the same cross sections for electromagnetic interactions. Hence, locally, they induce the same track structures and thus also the same DNA damage patterns; obviously, each track is unique due to stochastic effects, but we refer here to mean characteristics. However, notable kinematic effects arise at the cell nucleus level at low energies. The stopping power is identical for different isotopes of the given element so that their absolute rate of energy loss is the same. Nevertheless, the differing number of nucleons translates into variations in relative energy losses. As a consequence, longitudinally, the tracks of a lighter isotope vary faster than those of a heavier isotope of the same element. The energy deposited to the cell nucleus as a longitudinally integrated quantity thus does vary among isotopes, especially for hydrogen and helium but to a reduced extent also for heavier elements such as carbon. For instance, the energy deposition by tritons would have to be averaged over a 30 µm sized nucleus to provide the same results as protons over a 10 µm-sized nucleus.

Even when the induced DNA damage is scored as yields per Gy per Gbp, i.e., normalized to unit dose deposited to the nucleus, notable variations are present among diverse isotopes at low energies, especially for elements with low atomic numbers. As mentioned above, the track characteristics and hence also the induction of DNA damage are locally identical for different isotopes of the same element. However, the track characteristics and the induction of DNA damage vary longitudinally (i.e., along the penetration depth) due to gradually reduced velocity. These variations are very high for low-energy particles that stop within the nucleus, cf. the previously published results in which energy deposits and DNA damage induction by 0.25 MeV/u ^1^H, ^4^He and ^12^C were scored differentially in 200 nm slabs perpendicular to the particles’ direction of flight [34]. Due to kinematic effects, these variations in track characteristics and DNA damage induction along the penetration depth are more pronounced for lighter than for heavier isotopes of the same element. These variations manifest as differences in damage yields integrated over the cell nucleus.

Refined analytical formulas were provided that capture the simulation results. As discussed in detail previously [35,36], these analytical functions provide a means to extend the applicability of macroscopic transport codes to the endpoint of DNA damage induction. The refined formulas are valid over the whole studied energy range, from 0.5 GeV/u down to 0.25 MeV/u (or 0.1 MeV/u for H and He isotopes). Above 1 MeV/u, their results are virtually identical to the original formulas. Both the original and the refined formulas provide merely a fit to the simulation results, with isotope- and/or element-specific parameters, without aiming at providing a general theory or model of radiation-induced DNA damage. On the other hand, the underlying PARTRAC simulations do represent such a model, linking the variability in biological effects among diverse types of radiation to the differences in their track structures, describing the subsequent chemical and biological processes in a unique manner independently of the radiation type.

Taken together, the reported results indicate that at energies above 1 MeV/u or for elements heavier than helium, radiation-induced DNA damage, and hence likely also the subsequent biological effects such as chromosomal aberrations and cell killing, are virtually independent of the neutron number. This is in agreement with existing radiobiological evidence [21,22,23,24]. Specifically, in carbon radiotherapy or for radiation protection from AMS machines used in radiocarbon dating, knowledge of biological effectiveness gathered for ^12^C can also be applied to its lighter as well as heavier isotopes. Likewise, the biological effects of ^10^Be can be approximated by ^9^Be and those of ^10^B by ^11^B. On the other hand, notable variations are predicted for low-energy hydrogen and helium isotopes. In fact, these energies correspond to the Bragg peak region where most energy is deposited and also where the biological effectiveness is maximal, even when normalized per unit dose. For instance, 0.1 MeV/u tritons deposit 7.2 times more energy to the nucleus and induce 11.2 times more DSB sites per track, i.e., about 56% additional DSB sites per Gy in comparison with protons. The reported results may affect risk estimates and radiation safety measures for astronauts in deep space missions, in estimating the biological effectiveness of radiotherapy with ion beams as well as in boron-neutron and proton-boron capture therapies, where low-energy light elements and their diverse isotopes are produced in nuclear reactions.

## 4. Materials and Methods

PARTRAC [6,34] is a stochastic biophysical modelling tool for simulating the biological effects of ionizing radiation. It enables one to simulate the physics of interaction processes and tracks of photons, electrons, protons and heavier ions over energies relevant to medical and industrial applications [39]. A dedicated module traces subsequent chemical processes: the production of chemical species, their diffusion and mutual reactions, and the induction of damage to DNA [40]. DNA fragmentation patterns can also be analysed. A module is included to simulate DNA double-strand break repair by nonhomologous end-joining [41,42,43]. The models and their parameters have been extensively benchmarked against available experimental data and successfully applied in interpreting radiation quality-dependent biological effects [6]. PARTRAC has also been used to analyse initial events by which radiation modulates intercellular signalling [44,45,46].

In this work, particle tracks were simulated for ^1^H, ^2^H, ^3^H; ^3^He, ^4^He; ^6^Li, ^7^Li; ^9^Be, ^10^Be; ^10^B, ^11^B; and ^10^C, ^11^C, ^12^C, ^13^C and ^14^C with initial energies of 0.25–512 MeV/u (0.1–512 MeV/u for H and He isotopes). Results on the different types of associated DNA damage were obtained from human lymphocytes, following the previously developed methodology and simulation setup [34]. The particles were started perpendicularly from random positions in a circular source (diameter 10.92 µm) tangential to the spherical model of lymphocyte nuclei (diameter 10.00 µm), with a random rotation of the source with respect to the nucleus to avoid artefacts from the alignment of tracks with axes of the chromatin fibre model [34]. The starting energy values were log-equidistant, with a step corresponding to a factor of 2, with additional values for H and He isotopes at low energies, where the simulations resulted in maximal isotope specificity. The primary particles as well as secondary and higher-generation electrons were followed in a spherical region of interest (diameter 14.22 µm) concentric with the nucleus, filled with liquid water as a surrogate for biological medium, as performed previously [34]. The size of this simulated region was selected so as to include the source as well as most electrons potentially leaving and re-entering the nucleus upon multiple scattering. Note, however, that this setup does not guarantee electronic equilibrium conditions. Simulations with a full electronic equilibrium would be computationally extremely expensive, as mm-sized regions would be needed; 512 MeV/u ions may liberate approx. 1.4 MeV secondary electrons, whose range in water is about 7 mm [39].

Electromagnetic interactions (namely ionizations, excitations and charge transfer processes) were modelled as done standardly in PARTRAC [6,34]. At the same velocity (i.e., energy per nucleon), diverse isotopes of a given element were assumed to possess identical interaction cross sections, in agreement with physical theories and models stating that electromagnetic interactions are affected by the ion charge only, i.e., do not depend on the number of neutrons but protons only (reviewed in [37]). Charge states of hydrogen (H^+^ and H^0^) and helium (He^2+^, He^+^ and He^0^) were distinguished explicitly and dedicated cross sections for these states were used [47,48]. For heavier ions, the particle’s actual charge state was not traced, but the effective charge given by the ion energy was used to obtain cross sections by scaling those for hydrogen at the same velocity, as described in detail previously [34,49]. This approach correctly reproduces the stopping powers recommended by the ICRU for ions from Li to Ne [34]. In this work, this scaling approach was combined with the independence of electromagnetic interactions on neutron number and applied to diverse isotopes as well. As done standardly in PARTRAC and other track structure simulation codes, protons and heavier ions were assumed to travel along straight lines, neglecting their lateral scattering [6]. Likewise, nuclear reactions were not considered, as they occur much less frequently than electromagnetic interactions and hence may be neglected on the simulated scales.

Following the physics part of track structure simulations, energy deposits overlapping with the DNA model (including its hydration shell) were scored. Events outside the DNA were converted to chemical species. Their diffusion, mutual reactions, and attacks on the DNA structure were followed. Standard PARTRAC models and parameters were used, relevant for typical radiobiological experiments (under normal oxygenation, pH, salt concentrations, etc.), as reported previously [6]. In particular, the probability to induce a DNA strand break was assumed, for direct effects, to increase linearly with the energy deposited to a single sugar-phosphate group, from zero at and below 5 eV to unity at 37.5 eV and above, and for indirect effects, to correspond to 0.65 per hydroxyl radical attacking the deoxyribose moiety of DNA. Including both direct and indirect effects, DNA damage was classified as described previously [34]. Single-strand breaks (SSB, breaks on one DNA strand only), double-strand breaks (DSB, breaks on both strands within 10 base pairs, bp), DSB clusters (two or more DSB not separated by more than 25 bp) and DSB sites (a category including both isolated DSB and their clusters, but counting also a cluster of multiple DSB as a single DSB site) were scored.

Only initial DNA damage was considered; its subsequent repair was not simulated in this study. The simulation results are presented as yields per Gy per Gbp, in dependence on either initial particle energy, i.e., energy with which the particles are started from the source, or in dependence on the nucleus-averaged LET. Here, the deposited dose was obtained by scoring all energy deposits within the cell nucleus, divided by the nucleus mass (using the density of liquid water). The nucleus-averaged LET was obtained from the dose–particle fluence relationship. For simplicity and to facilitate applications of the present results in studies using other codes, when scoring energy deposits and calculating the dose and LET, the volume of the whole nucleus was taken, even for low-energy particles that actually penetrate only a part of the nucleus and stop inside it. For consistency with the common use in the field, the terms ‘dose’ and ‘LET’ are used throughout this work for quantities at the level of the cell nucleus (10 µm diameter sphere), although microdosimetry terms, such as specific energy and lineal energy, would be more appropriate [50].

Per energy value and for each isotope considered, at least 16,000 hydrogen, 8000 helium, 1280 lithium or 640 beryllium, boron or carbon ions were simulated. The numbers of impinging particles were selected to guarantee that statistical errors (standard deviations) of the estimated DNA damage yields calculated on the basis of Poisson distributions were sufficiently small, below 2% for total DSB and DSB sites. This ensured SSB errors were within 0.6%. DSB clusters, which are notably less frequent, were subject to larger uncertainties, 6% on average but up to 24% for sparsely ionizing high-energy ions.

The results of isotope-specific PARTRAC simulations were compared with previously published analytical formulas [36]. For H and He isotopes, refined fits were obtained with Matlab (The MathWorks Inc., Natick, MA, USA), using the formula

(1) $$Y=p_{0}+\frac{p_{1}E^{p_{2}}}{{\left(1+\exp(p_{3})E^{p_{2}+p_{4}}\right)}^{p_{7}}}{\left(1+\exp(p_{5})E^{p_{6}}\right)}^{p_{8}}$$

where *Y* denotes the DNA damage yields per Gy per Gbp, *E* stands for the particles’ starting energy in MeV/u, and *p*_0–8_ are fit parameters. The same formula was also used for the SSB damage class, which was not included in the previous publication [36].

## Figures and Tables

**Table 1:** Parameters of analytical functions (Equation 1) used to represent the simulation results.

Damage Class	Species	*p*_0_ *	*p*_1_	*p*_2_	*p*_3_	*p*_4_	*p*_5_	*p*_6_	*p*_7_ **	*p*_8_ **
SSB	^3^H	156	−24.93	−0.3641	−6.929	−6.011	−6.255	−6.115	1	1
	^2^H	156	−25.31	−0.3800	−6.719	−7.741	−6.305	−7.999	1	1
	^1^H	156	−25.94	−0.3725	−4.438	−8.884	−4.152	−9.307	1	1
	^4^He	156	−49.45	−0.4011	−1.353	−3.763	−0.7219	−3.719	1	1
	^3^He	156	−48.02	−0.3839	−0.6727	−4.131	−0.07540	−4.142	1	1
	Li	156	−41.32	−0.2756	−1.375	−1.316	0.2535	−0.9409	1	1
	Be	156	−22.54	−0.1273	−1.359	−1.136	1.388	−0.6218	1	1
	B	156	−4.762	0.1012	−1.214	−1.121	3.253	−0.6463	1	1
	C	156	−3.197	0.1951	−0.2466	−1.018	4.076	−0.7828	1	1
DSB sites	^3^H	6.8	632.3	2.302	4.961	0.7071	−4.408	−2.148	1	1
	^2^H	6.8	321.9	2.529	4.327	0.6768	−2.627	−1.894	1	1
	^1^H	6.8	69.50	2.468	3.406	0.4781	0.2036	−0.9876	1	1
	^4^He	6.8	6.288	0.3915	0.06198	0.8175	0.7250	1.052 × 10^−4^	1	1
	^3^He	6.8	7.374	0.5449	0.3136	0.7952	0.7243	−2.555 × 10^−5^	1	1
DSB clusters	^3^H	0.07	61.58	3.110	6.153	1.421	−3.013	−2.496	1	1
	^2^H	0.07	9.363	3.088	4.606	0.9136	−0.6496	−2.212	1	1
	^1^H	0.07	0.2697	3.691	3.106	0.2513	2.557	−2.827	1	1
	^4^He	0.07	4.605	1.721	1.803	1.089	0.07841	−1.110	1	1
	^3^He	0.07	1.791	1.989	1.407	0.9043	1.159	−1.284	1	1
DSB	^3^H	6.8	37.22	0.6415	7.099	4.083	−5.500	2.588	0.2926	0.1174
	^2^H	6.8	42.81	0.8008	5.279	3.209	0.8288	1.586	0.5030	0.3777
	^1^H	6.8	473.1	2.028	1.682	−0.3444	−0.9622	4.825	2.515	0.3132
	^4^He	6.8	10.56	0.6902	1.331	0.8017	1.317	3.769 × 10^−5^	0.9350	0.9792
	^3^He	6.8	9.604	0.6996	1.425	1.220	1.318	7.779 × 10^−5^	0.7288	0.8862

* Parameter *p*_0_ determining the high-energy damage yields was taken from Ref. [36]. ** For SSB, DSB sites and clusters, parameters *p*_7_ and *p*_8_ were not used (i.e., fixed at unity, as they serve as exponents in Equation (1)).

## Data Availability

The data presented in this study are available on request from the corresponding author.
